# BmTGIF, a *Bombyx mori* Homolog of *Drosophila* DmTGIF, Regulates Progression of Spermatogenesis

**DOI:** 10.1371/journal.pone.0047861

**Published:** 2012-11-09

**Authors:** Pengjie Zhang, Guangli Cao, Jie Sheng, Renyu Xue, Chengliang Gong

**Affiliations:** 1 School of Biology and Basic Medical Science, Soochow University, Suzhou, People’s Republic of China; 2 National Engineering Laboratory for Modern Silk, Soochow University, Suzhou, People’s Republic of China; University of Maryland School of Medicine, United States of America

## Abstract

TG-interacting factor (TGIF) in *Drosophila* consists of two tandemly-repeated genes, *achintya* (*Dmachi*) and *vismay* (*Dmvis*), which act as transcriptional activators in *Drosophila* spermatogenesis. In contrast, TGIF in humans is a transcriptional repressor that binds directly to DNA or interacts with corepressors to repress the transcription of target genes. In this study, we investigated the characteristics and functions of BmTGIF, a *Bombyx mori* homolog of DmTGIF. Like DmTGIF, BmTGIF is predominantly expressed in the testes and ovaries. Four alternatively spliced isoforms could be isolated from testes, and two isoforms from ovaries. Quantitative polymerase chain reaction indicated *BmTGIF* was abundantly expressed in the testis of 3rd instar larvae, when the testis is almost full of primary spermatocytes. The results of luciferase assays indicated that BmTGIF contains two adjacent acidic domains that activate the transcription of reporter genes. Immunofluorescence assay in BmN cells showed that the BmTGIF protein was located mainly in the nucleus, and paraffin sections of testis showed BmTGIF was grossly expressed in primary spermatocytes and mature sperms. Consistent with the role of DmVis in *Drosophila* development, BmTGIF significantly affected spermatid differentiation, as indicated by hematoxylin-eosin staining of paraffin sections of testis from *BmTGIF*-small interfering RNA (siRNA)-injected male silkworms. Co-immunoprecipitation experiments suggested that BmTGIF interacted with BmAly, and that they may recruit other factors to form a complex to regulate the genes required for meiotic divisions and spermatid differentiation. The results of this analysis of BmTGIF will improve our understanding of the mechanism of spermatid differentiation in *B. mori*, with potential applications for pest control.

## Introduction

Homeodomain proteins were first discovered in *Drosophila* where they are responsible for regulating segment identity. They have subsequently been found in diverse species and multiple organisms as transcription factors, such as members of the HOX family, where they play important roles in the regulation of tissue-specific and developmental processes [1−5]. The homeodomain comprising about 60 amino acids is a conserved module in proteins, and consists of three helices [1], [6].The first two helices are usually involved in protein-protein interactions, which can alter the DNA-binding specificity [7−9], while the third helix, which regulates DNA-binding, usually binds to relatively simple DNA sequences. The third position of the conserved WF_N sequence in helix 3 has been shown to be important for determining the DNA-binding sequence specificity. PBC, HOX and Prep1 proteins contain a glycine, glutamine and isoleucine amino acid at this position, respectively [10−12]. Homeodomain proteins can be classified into different groups based on the homeodomain sequence, the flanking amino acids, and the other regions. One such group is the TALE group [1], the superfamily homeodomain proteins of which are characterized by the presence of three amino acids between helix 1 and helix 2, resulting in a 63-amino acid homeodomain, instead of the more typical 60. The three amino acids in the PBC TALE subgroup are Leu Ser Asn [13], compared to Arg Tyr Asn in the TG-interacting factor (TGIF) subgroup [14]. Although there are three amino acids between the first two helices, the insertion is unlikely to affect DNA-binding, and may conversely play a role in protein-protein interactions [13], [15], [16]. The TALE homeodomain proteins are an ancient family represented in organisms as diverse as yeast and humans. They act as transcription factors, usually together with other transcription factors, and exert their effects on the expression of required genes by both activating and repressing their transcriptional activities [7], [12], . Members of this superfamily of proteins include yeast MATα2 [22], maize Knotted-1 [23], *Caenorhabditis elegans* ceh-25, the fly Iroquois complex genes [24], the human protooncogene PBX 1 [19], [21], and the transcription factors TGIF [10] and MEIS 1 [20].

TGIF, which has been identified as a transcription factor in many distinct pathways, forms a subgroup of TALE homeodomain proteins. TGIF was first identified as a transcriptional repressor that competed with retinoid receptors for binding to the retinoid response element (RXRE) of the cellular retinol binding protein II (CRBP II) gene, resulting in reduced expression of the CRBP II gene. The consensus sequence responsible for binding to TGIF has been determined *in vitro* [10]. Apart from binding to the DNA-binding sequence alone via its homeodomain, TGIF has also been shown to work with Smad proteins, which are critical transcription factors, in response to transforming growth factor β signaling, to repress the transcriptional activity of target genes [10], . In humans, mutations in TGIF are associated with holoprosencephaly (HPE), a severe genetic disease affecting craniofacial development [30], [31]. Recent studies in *Drosophila* have demonstrated that dTGIF is transcriptional activator of genes required for spermatogenesis. Loss of function mutations in dTGIF resulted in arrest of testis development at the primary spermatocyte stage, prior to entering meiotic divisions and spermatid differentiation, and also in delayed egg laying [14], [32], [33].

Spermatogenesis represents a dramatic example of developmental control of gene expression, and many genes required for this process are only switched on in this cell type. Some are germline-specific homologs of ubiquitously-expressed genes, e.g., *β2 tubulin* and the mitochondrial fusion protein *fzo* [34], [35], while others are spermatogenesis specific genes, e.g., *protamine* and *gonadal* [36], [37]. *Drosophila* spermatogenesis is maintained from the larval stage to adult life, and all stages of differentiation occur in a single adult testis [38]. Also in *Drosophila*, several reports have shown that meiotic divisions and spermatid differentiation are mediated by the activity of meiotic-arrest genes, although the initiation of spermiogenesis is independent of meiotic divisions. A few meiotic-arrest-class genes have been studied to date in *Drosophila*, including *always early* (*aly*), *cannonball* (*can*), *meiosis I arrest* (*mia*), *spermatocyte arrest* (*sa*), *no-hitter* (*nht*), *cookie monster* (*comr*), *achintya/vismay* (*achi/vis*), *matotopetli* (*topi*) and *tombola* (*tomb*) [33], .

In the silkworm *Bombyx mori*, spermatogenesis has been well studied at the ultrastructural level [45], [46]. Six mitotic divisions of a spermatogonial cell produce a cyst of 64 primary spermatocytes,which immediately undergo premeiotic S stage. They then enter an extended G2 period, following by meiotic divisions and consequently generates a cyst with 256 spermatids. Although the molecular mechanism of *Drosophila* spermatogenesis has been extensively investigated, the situation in Lepidoptera is less well known [47], [48]. The genome sequence of *B*. *mori*, as a model of Lepidopteran insect, has been well established [49], [50], and many genes related to growth, development, metamorphosis, immunologic response, and fibroin synthesis have been well studied. However, little is known regarding genes related to spermatogenesis. Like *Drosophila*, spermatogenesis in *B*. *mori* also occurs throughout larval and adult life, from spermatogonia in newly-hatched silkworms to mature sperm in the moth. Unfortunately, the mechanisms that regulate cell-cycle progression and cellular differentiation of testis development in *B*. *mori* have not been elucidated in detail. In this study, we characterized *BmTGIF*, a homolog of the *Drosophila* meiotic-arrest gene *DmVis*, and demonstrated that *BmTGIF* also acts as a meiotic-arrest gene regulating meiotic progressions and spermatid differentiation.

## Materials and Methods

### Ethics Statement

Kunming mice were obtained from the Center for Experimental Animal, Soochow University (Certificate No. 20020008, Grade II), and the animals tolerated the subcutaneous injection well, no deaths occurred in the procedure. The Ethical Committee of Soochow University specifically approved the current study.

### Insects and Cell Culture

The non-diapausing Dazao strain of *B*. *mori* was used for RNA interference studies. Larvae were reared on fresh mulberry leaves at 25°C. The BmN cell line derived from the ovary of silkworms was cultured at 25°C in TC-100 insect medium (US Biological) supplemented with 10% (v/v) heat-inactivated fetal bovine serum (Gibco-BRL, Gaithersburg, USA).

### RNA Isolation and cDNA Synthesis

Total RNA was isolated from the testes of fifth instar silkworm larvae using a total RNA Isolation Kit (TaKaRa, DaLian, China), followed by treatment with DNaseI to remove possible contamination with genomic DNA. cDNA was synthesized by PrimeScript™ Reverse Transcriptase (TaKaRa), following standard instructions.

### Cloning and Sequencing of the *BmTGIF* Gene

The *BmTGIF* gene-specific primers *BmTGIF*-1 and *BmTGIF*-2 contain BamHI and EcoRI restriction sites at their 5′ ends, respectively. The primers were designed according to the hypothetical *BmTGIF* gene cDNA sequence, which was obtained through *in silico* cloning based on DmVis protein sequences (GenBank accession no. AAO41402), using the tBLASTn program (http://www.ncbi.nlm.nih.gov/blast). Polymerase chain reaction (PCR) was carried out using the synthesized cDNA as a template and the paired primers *BmTGIF-1*/*BmTGIF*-*2*. The PCR product was subjected to agarose gel electrophoresis and the recombinant plasmids were sequenced after the recovered PCR product was cloned into the vector pMD19-T (TaKaRa).

PCR was also carried out with primers *BmTGIF-1/BmTGIF-3* to analyze alternatively-spliced isoforms of *BmTGIF*. The sequences were deposited in GenBank.

### Bioinformatics Analysis

A homology search and multiple alignments were carried out using BLAST (http://www.ncbi.nlm.nih.gov/BLAST) and the ClustalW software program [51], respectively. A phylogenetic tree was constructed using MEGA 5.0 and the neighbor-joining (NJ) method [52], based on the TGIF entire protein sequences retrieved from GenBank. The validity of the various branches of the trees was tested by bootstrapping using 500 replicates.

### BmTGIF-L Expression in Escherichia coli and BmTGIF-L Antibody Preparation

The *BmTGIF* PCR product (1.1 kb) (*BmTGIF-L*) was digested with BamHI/EcoRI and ligated with the expression vector pET-28a(+) (Novagen) to generate the recombinant plasmid pET-28a(+)-*BmTGIF-L*. Fusion proteins were expressed in *E*. *coli* strain BL21. The recombinant protein purified using Ni-NTA agarose (Qiagene, Shanghai, China) was used to immunize Kunming mice (Soochow University, Suzhou, China) by subcutaneous injection. The prepared antibody was then identified by Western blotting.

### Sodium Dodecyl Sulfate-polyacrylamide Gel Electrophoresis (SDS-PAGE) and Western Blotting

The bacterium transformed with pET-28a (+)-*BmTGIF* was mixed with 2 × SDS loading buffer (0.1 mol/L TrisCl, 0.2 mol/L dithiothreitol, 4% SDS, 20% glycerol, 0.2% bromophenol blue, 4% β-mercaptoethanol) and boiled in 100°C water for 5 min. After centrifugation at 12,000 *g* for 3 min, the supernatant was electrophoresed on acrylamide gels. The stacking and separating gels were 5% (v/v) and 12% (v/v), respectively. The gel for protein staining was treated with Coomassie Brilliant Blue R250. The gel for Western blotting was transferred to a polyvinylidene fluoride membrane using an electrophoretic transfer cell. Western blotting was then performed using a prepared mouse anti-BmTGIF-L primary antibody and a horseradish peroxidase (HRP)-conjugated goat anti-mouse IgG (Biosynthesis Biotechnology, Beijing, China).

Western blotting was also used to detect BmTGIF in testes and ovaries, about 20 testes and ovaries were independently dissected using protein lysis buffer, the lysates of them were then put into 2×SDS sample buffer for SDS-PAGE followed by western blotting detection.

### Expression Analysis

For *BmTGIF* expression profile analysis, RNA was extracted from heads, hemocytes, midgut, fat bodies, malpighian tubules, silk glands, testes and ovaries of *B. mori*. cDNAs were synthesized by reverse transcription with 1 µg of this RNA, using PrimeScript™ Reverse Transcriptase (TaKaRa). Before reverse transcription, the mRNA samples were fully digested with RNase-free DNaseI to avoid genomic DNA contamination. Primers *ReTGIF-1* and *ReTGIF-2* were designed based on the cDNA sequences. *ReBmactin3-1* and *ReBmactin3-2* were used as primers for amplifying the housekeeping gene *B*. *mori actin A3*, used as an internal control. PCR was carried out according to the following program: one cycle at 95°C for 3 min; 35 cycles at 94°C for 30 s, 59°C for 30 s and 72°C for 30 s; one final cycle at 72°C for 10 min. The product was then analyzed by agarose gel electrophoresis.

Quantitative PCR (Q-PCR) was used to determine the expression levels of *BmTGIF*-L in the testes at different stages using primers *ReTGIF-1*/*ReTGIF-2* and *ReBmactin3-1/ReBmactin3-2*. A 20-µl volume containing 0.2 µg cDNA, 5 pmol of each primer, and 10 µL of SYBR Green Real-time PCR Master Mix (ABI, USA) was used for Q-PCR reaction. Q-PCR was carried out using a real-time reverse transcription (RT)-PCR System (ABI 7300) according to the following program: one cycle at 50°C for 2 min; one cycle at 95°C for 10 min; 40 cycles at 95°C for 15 sec, 60°C for 1 min; one final cycle for dissociation at 95°C for 15 sec, 60°C for 30 sec and 95°C for 15 sec. This experiment was repeated three times.

To determine the relative amounts of the various alternatively-spliced isoforms in the testes at the beginning of the fifth instar larvae, Q-PCR was carried out using different primer pairs of FJ643616, FJ913885, FJ913886, and GQ120180.

### Luciferase Reporter Assay

A plasmid pSK-IE(S) containing the *B. mori* nucleopolyhedrovirus *ie-1* promoter (GenBank accession No. AY616665) was used to generate luciferase assay plasmids pIE-A1, pIE-A2 and pIE-A1+2. In these plasmids, the protein sequence corresponding to the acidic domains 1 (A1, at position 190–268 of the longest BmTGIF isoform), 2 (A2, at position 282–360 of the longest BmTGIF isoform) and 1+2 (A1+A2) of BmTGIF that containing the initiation codon ATG and stop codon TAG. A luciferase gene (*luc*) was cloned into the plasmid pSK-IE containing the *ie-1* promoter with the TGIF target sequence CTGTCAA to generate the firefly luciferase reporter plasmid pIE-luc. pSK-IE was used as a blank control, and the internal control luciferase plasmid pRL-TK [53], in which the *Renilla* luciferase gene (*Rluc*) is driven by the herpes simplex virus thymidine kinase (HSV-TK) promoter.

BmN cells were cotransfected with the plasmid pIE-luc (1 µg) and the other above-mentioned plasmids (2 µg) using Lipofectamine (Invitrogen). At 60 h post-transfection, the cells were harvested and the luciferase activities were measured using a luminometer (GLOMAX-MULTI JR, Promega) and a Dual-Lucy Assay Kit (Vigorous, China). Various doses of plasmid pIE-A2 (0.5, 1.0, 1.5 and 2.0 µg) were tested to detect dose-dependent effects of BmTGIF on luciferase activity. All luciferase assay experiments were performed with co-transfection of *Renilla* (pRL-TK) (100 ng) as an internal control. Each experiment was performed in triplicate, and the data represent means ± SD of three independent experiments after normalization to *Renilla* activity.

### RNAi

The *BmTGIF*-specific siRNA-4 (sense: 5′ ccguuacacuacacgaucutt 3′, antisense: 5′ agaucguguaguguaacggtt 3′) which targeted exon 3 of *BmTGIF*-L, was synthesized by Gima Corporation (Shanghai, China). A total number of 40 silkworm larvae were injected with 1 µg of *BmTGIF-*siRNA-4 per larva at the first day of the third, fourth and fifth instars, and reared on fresh mulberry at 25°C. The expression level of *BmTGIF*-L in the testes of injected larvae at the end of the fifth instar was estimated by Q-PCR. Meanwhile, Blank controls that raised normally and NC-siRNA (sense: 5′ uucuccgaacgugucacgutt 3′, antisense: 5′ acgugacacguucggagaatt 3′) were tested. Besides detecting the *BmTGIF*-L mRNA level among them, Western blotting was also used to detect the *BmTGIF*-L protein production.

### Immunofluorescence and Histochemical Observations

BmN cells and paraffin sections of testes at pupal stage (10 days after pupation) were fixed with 4% paraformaldehyde for 15 min, then rinsed with 0.01 M PBST (0.05% Tween-20 in 0.01 M phosphate-buffered saline) and incubated with a mouse anti-BmTGIF-L antibody at 4°C overnight. At the same time, BmN cells/paraffin sections of testes were incubated with pre-immune antiserum as a negative control. After rinsing three times with 0.01 M PBST, the BmN cells/paraffin sections were incubated with fluorescein isothiocyanate (FITC)-conjugated goat anti-mouse IgG (Tiangen) at 37°C for 1 h, then observed by fluorescence microscopy after removing the non-combined FITC-conjugated goat anti-mouse IgG and staining with 4',6-diamidino-2-phenylindole (DAPI).

For histochemical observations, the larvae were injected with 1 µg of *BmTGIF*-siRNA-4 per larva at the beginning of the third, fourth and fifth instars. The testes were dissected from the injected larvae at the beginning, at the end of the fifth instar larvae, and at 10 days after pupation, fixed in 4% paraformaldehyde and embedded in paraffin. The sections were then stained with hematoxylin-eosin (HE) solution for observation. Uninjected blank controls were prepared simultaneously.

### Co-immunoprecipitation

Approximately 30 testes were dissected in 100 ml RIPA buffer(50 mM Tris pH 7.5, 150 mM NaCl, 20 mM MgCl_2_, 0.5% NP40, and 1 mM PMSF) containing a protease inhibitor cocktail (Roche) and 2.5 mM Na_3_VO_4_ (Sigma). Testes extracts were obtained by removing cell debris by centrifugation at 12,000 *g* for 3 min, and the lysates were then precleared with pre-immune antiserum (Kunming mice) for 30 min, and protein G Plus/Protein A Agarose for a further 30 min. Precelared proteins and BmTGIF antibody were mixed at 1∶100 dilution and incubated at 4°C overnight, followed by addition of 50 µl protein G Plus/Protein A Agarose Suspension (Calbiochem, Germany) and incubation for an additional 3 h. Samples were then washed three times with washing buffer (50 mM Tris-HCl, pH 8.0, 150 mM NaCl, 1% NP-40, 0.5% sodium deoxycholate, and 0.1% SDS) and the immune complexes were mixed with sample-loading buffer (0.1 mol/L TrisCl, 0.2 mol/L dithiothreitol, 4% SDS, 20% glycerol, 0.2% bromophenol blue, 4% β-mercaptoethanol) for 5 min at 100°C and analyzed by SDS–PAGE. Western blotting was performed with anti-BmAly antibody (1∶100) and secondary HRP-conjugated antibody (Amersham Biosciences). Conversely, testes extracts were incubated with BmAly antibody (saved in our laboratory) for SDS-PAGE. Western blotting with BmTGIF antibody was performed simultaneously. For controls, the protein samples were obtained by mixing testes extracts with pre-immune antiserum (Kunming mice) and protein G Plus/Protein A Agarose. Western blotting was performed as same as above mentioned.

## Results

### Splicing of *BmTGIF* gene in Testes and Ovaries

The *BmTGIF* gene sequence was obtained by *in silico* cloning. Primer pairs *BmTGIF-1/BmTGIF-2* were designed according to the hypothetical *BmTGIF* gene cDNA sequence, and used for cloning the *BmTGIF* gene. Three specific products (1.1, 0.47 and 0.32 kb) were amplified from testes cDNA, but only one product (1.1 kb) from ovary cDNA. Sequencing results showed that the ovary sequence was the same as the largest fragment from the testes, 1083 bp (FJ643616), encoding a protein (BmTGIF-L) with 360 amino acid residues. The two smaller fragments of 315 (FJ913885), and 468 (FJ913886) bp encoded proteins BmTGIF-MS1 and BmTGIF-MS2, of 104 and 155 amino acid residues, respectively. To investigate other possible isoforms, an internal primer *BmTGIF-3* was designed according to the BmTGIF-L sequence. PCR was carried out with the primer pairs *BmTGIF-1/BmTGIF-3* and a 0.24-kb fragment was amplified from sexual gland cDNA. Sequencing showed that this fragment was 240 bp (GQ120180), and the sequence differed from the sequences mentioned above.

**Figure 1:**
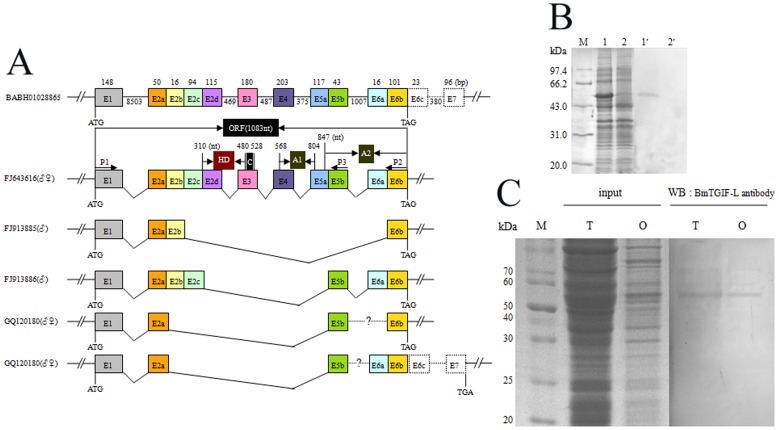
The gene structure and expression patterns of *BmTGIF* in sexual glands. (A) The *BmTGIF* gene structure and alternative splicing products determined at mRNA level by complete sequencing of the following cDNAs: FJ643616 (*BmTGIF*-L), FJ913885 (*BmTGIF*-MS1), FJ913886 (*BmTGIF*-MS2), and GQ120180 (*BmTGIF*-S). The numbers are indicated above, the distances are indicated below. The juctions of alternative spliced variants are marked as indicated. The gene of *BmTGIF* is located at a contig BABH01028865 of *B. mori* genome, predicted E6c and E7 was obtained by blasting FJ643616 with ESTs of *B. mori*, resulting three ESTs (CK564126, DC564285, DB674187) could be assembled together. The predicted E6c and E7 are boxed with dashed line to distinguish from the sequenced exons. ORF of *BmTGIF* was indicated in the schematic, consisting of six exons as a molecular weight of 1083 nt. HD (homeodomain) locates at the position of 310 to 480 nt, which spans parts of E2 and E3. The primers used to detect the alternative splicing products was named P1 (*BmTGIF*-1), P2 (*BmTGIF*-2), and P3 (*BmTGIF*-3) as indicated in Table 1, locating at 5′ terminal of exon 1, 3′ terminal of exon 6, and 3′ terminal of exon 5, respectively. In testes (♂), all the four spliced variants were found; while in ovaries (♀), only FJ643616 (*BmTGIF*-L) and GQ120180 (*BmTGIF*-S) were detected. As for GQ120180, there maybe two spliced isoforms existed, by which exon E5b ligated to end, the two predicted alternative spliced ways for GQ120180 were marked with dashed line. (B) Prokaryotic expression and Western blotting for BmTGIF polyclonal antibody specificity. M, protein marker; lane 1, SDS-PAGE of Pet-28a (+)-*BmTGIF* expressed in *E. coli* BL21, a specific band was detected at about 52 kDa; lane 2, *E. coli* strain BL21 transformed with Pet-28a (+); lane 1′, Western blotting was performed with primary antibody mouse anti-BmTGIF-L followed by secondary antibody HRP-conjugated goat anti-mouse IgG. A prominent band corresponding to lane 1 occurred; lane 2′, Western blotting corresponding to lane 2. (C) The alternative splicing products were determined at protein level by western blotting using BmTGIF-L antibody. In both testes and ovaries, only the product of *BmTGIF*-L is observed that appears to migrate slightly slower than the predicted 40 kDa, about 55 kDa; the reason for this maybe glycosylation or other modifications after translation.

To analyze the protein products derived from *BmTGIF* we used the anti-BmTGIF-L antibody in western blotting experiments. A band with apparent molecular mass of ∼55 kDa (BmTGIF-L) was detected in both the lysates of testes and ovaries (Fig. 1C). However, other spliced isoforms were not detected in the testes and ovaries, this maybe ascribed to the expression level of other spliced isforms that was too low to be detected.

**Table 1:** Primer pairs used for the study of BmTGIF in this paper.

	Forward primer	Reverse primer
**For cloning and** **sequencing of ** ***BmTGIF***	ctggatccatgttacctcccacggccggtctc(*BmTGIF*-1)	**ctgaattcctagacgggcagctcgtcggcttcc(** ***BmTGIF*** **-2)**
**For alternative** **splicing of ** ***BmTGIF***	ctggatccatgttacctcccacggccggtctc(*BmTGIF*-1)	**ctgaattcctagacgggcagctcgtcggcttcc(** ***BmTGIF*** **-2)** **gcagccgtttcctgagcgggtg(** ***BmTGIF*** **-3)**
**Re** ***TGIF***	cggagctgatgttgagaatg	**accgcactggaggagtagcc**
**ReBmactin3**	ctgcgtctggacttggc	**cgagggagctgctggat**
**ReFJ643616**	cggagctgatgttgagaatg	**accgcactggaggagtagcc**
**ReFJ913885**	acagacggcgaggcgggcga	**ctagacgggcagctcgtcgg**
**ReFJ913886**	gaacctcgcacatcttccgc	**acggcggtctccaccagcag**
**ReGQ120180**	gagagcggcacatcttccgc	**tcatagatgagagtggtggtac**
**Acid domain 1**	tccaagcttatggtcgttgtcgcttcttg	**acgctcgagttatctgacagcgggatgtatctc**
**Acid domain 2**	**tccaagcttatggccgtggagggcgac**	**acgctcgagctagacgggcagctcgtc**

Blast search showed that FJ643616 was overlapped with EST sequences (CK564126, DC564285, DB674187) and followed by 119 bp of 3′ untranslated region. The *BmTGIF* cDNA sequence with 3′ untranslated region was compared to the genomic sequence of *B. mori* using the BlastN program (http://blast.ncbi.nlm.nih.gov/Blast.cgi). The results showed that the genomic sequence of *BmTGIF* was completely located in a genomic DNA fragment (BABH01028865) (Fig. 1A); The open reading frame (ORF) of the longest isoform *BmTGIF*-L (FJ643616) has six exons, both exon 6c and exon 7 were untranslated sequence; while exons 3–5 are in the sequence FJ643616, but not in the sequence of *BmTGIF*-MS1 (FJ913885). ORF of *BmTGIF*-MS1 was composed of exon 1, exon 2a, exon 2b and exon 6b in FJ643616. Exons 3–4 in the sequence of FJ643616 were not in *BmTGIF*-MS2 (FJ913886), while exon 2 of *BmTGIF*-MS2 was composed of exon 2a, exon 2b and exon 2c in FJ643616, exon 3 and 4 of *BmTGIF*-MS2 were exon 5b and exon 6 in FJ643616, respectively. In the *BmTGIF*-S sequence, its 5′ terminal sequence was composed of exon 1, 2a and 5b of sequence FJ643616, but the splicing form at 3′ terminal was undetermined, if exon 5b was followed by exon 6b, the deduced ORF of *BmTGIF* was 342 bp in length, encoding a protein with 113 amino acid residues, if exon 5b was followed by exon 6, the stop codon TGA was appeared at 3′ terminal of exon 7, the deduced ORF of *BmTGIF* was 477 bp in length, encoding a protein with 158 amino acid residues.

### Protein Expression in *E. coli* and BmTGIF Antibody Preparation

*E*. *coli* transformed with pET-28a(+)-*BmTGIF* were induced with isopropyl-β-D-thio-galactoside for a further 6 h at 37°C after reaching the logarithmic phase. Harvested bacteria were subjected to SDS-PAGE to detect BmTGIF-L expression, and a specific band of about 52 kDa representing 6×His-BmTGIF fusion protein was detected (Fig. 1B, lane 1), indicating that BmTGIF was correctly expressed in *E*. *coli*. The recombinant protein was then purified using Ni-NTA agarose (Qiagene), and was used to immunize mice to prepare polyclonal antibody.

Western blotting was used to assess the efficacy of the prepared mouse anti-BmTGIF antibody. As expected, a specific band representing the 6×His-BmTGIF fusion protein was also detected at the position of the recombinant protein (Fig. 1B, lane 1′), indicating that the prepared antibody was suitable for further studies.

### BmTGIF-L is a TALE Homeodomain Protein

Sequence comparisons of BmTGIF proteins showed that the 66 (exon 1, 2a) amino acid residues at the amino-terminal were conserved among the four BmTGIF isoforms, while 32 (exon 6b) amino acid residues at the carboxy-terminal were conserved among three BmTGIF isoforms (BmTGIF-L, BmTGIF-MS1 and BmTGIF-MS2).

**Figure 2:**
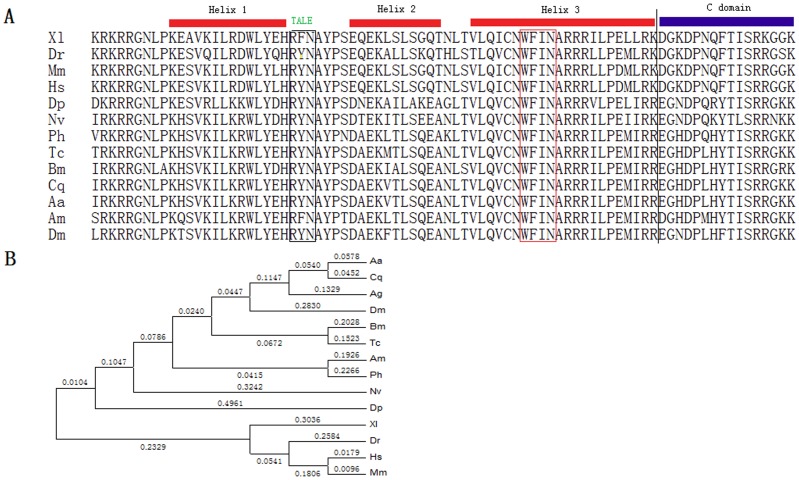
Sequence analysis of BmTGIF. (A) Sequence comparison showed that the homeodomain and adjacent carboxy-terminal region are conserved among the tested species. Vertical line indicates the carboxy-terminal limit of the homeodomain; the TALE amino acids, RYN, which are characteristic of the TGIF families are indicated by a black box; the DNA-binding-determining residue between F and N in WFIN are indicated by a red box. (B) The full amino acid sequences of TGIF were used to construct the phylogenetic tree using a NJ program with bootstrapping using 500 replicates. The bootstrap values are shown on each internal node. Bm, *Bombyx mori*; Dm, *Drosophila melanogaster*; Nv, *Nasonia vitripennis*; Ag, *Anopheles gambiae*; Am, *Apis mellifera*; Hs, *Homo sapiens*; Mm, *Mus musculus*; Dr, *Danio rerio*; Aa, *Aedes aegypti*; Cq, *Culex quinquefasciatus*; Tc, *Tribolium castaneum*; Ph, *Pediculus humanus corporis*; Dp, *Daphnia pulex*; Xl, *Xenopus laevis*.

Structural analysis showed that there were three helices (positions 95–160), a carboxy terminal to the helices (positions 161–176) (Fig. 2A), and two acidic domains (positions 190–268 and 282–360) in the BmTGIF-L protein, while three amino acid residues (RYN), a characteristic sequence of TALE superfamily homeodomain proteins, were also located between helix 1 and helix 2 (Fig. 2A). Moreover, WF_N, the key residue between F and N residues that is usually considered to be important in determining the DNA-binding-sequence specificity [11], [54], [55], was present in helix 3 (Fig. 2A).

The deduced amino acid sequence of BmTGIF-L was aligned with those of 13 other species. BmTGIF-L shared 38.5, 41.9, 36.1, 34.0, 52.0, 45.4, 38.8, 32.3, 31.5, 30.2, 30.5, 33.3 and 32.5% similarity with TGIF of *Aedes aegypti* (XP_001653660), *Culex quinquefasciatus* (XP_001849992), *Anopheles gambiae* (XP_308755), *D. melanogaster* (NP_725182), *Tribolium castaneum* (NP_001153689), *Apis mellifera* (XP_001122713), *Pediculus humanus corporis* (XP_002425861), *Nasonia vitripennis* (NP_001153690), *Daphnia pulex* (EFX88970), *Xenopus laevis* (NP_001087637), *Danio rerio* (NP_955861), *Homo sapiens* (NP_068581) and *Mus musculus* (NP_775572), respectively. Although the sequence identities of the TGIF proteins from different species were not high, three conserved helixes and one adjacent C-domain were prominent (Fig. 2A).

Based on the high degree of similarity of the homeodomain and the key amino acid for determining DNA-binding specificity between BmTGIF and other homologs, we speculate that BmTGIF may have the same function as DmTGIF, and may bind to the same DNA sequence.

To investigate the evolutionary relationship between TGIF proteins in different species, a phylogenetic tree was constructed using the NJ method, based on the amino acid sequence of TGIF. The *TGIF* gene from different insect species gathered together, while the vertebrate genes formed another group (Fig. 2B).

### *BmTGIF* was Expressed in Hemocytes and Head, as well as Testis and Ovary

**Figure 3:**
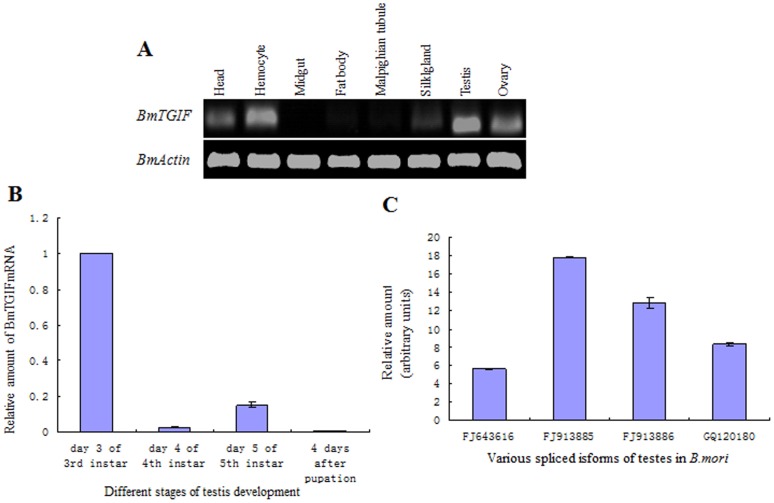
Expression profiles of *BmTGIF*. (A) RT-PCR analysis of *BmTGIF*-mRNA in various *B. mori* tissues. Total RNA from each tissue was used for cDNA synthesis. PCR products amplified with gene-specific primers (Table 1) for *BmTGIF* and *Bmactin A3* were analyzed on 1% agarose gels and visualized by ethidium bromide staining. (B) Q-PCR analysis of *BmTGIF* mRNA at different stages of testis development. Testes were dissected from silkworms at the times indicated, and cDNA was synthesized as described in (A). The *BmTGIF* expression levels were normalized against the housekeeping cytoplasmic β-actin expression levels. (C) The relative amounts of alternatively-spliced isoforms were detected by Q-PCR using specific primers (Table 1). cDNAs were synthesized using total RNA extracted from testes at day 5 of fifth instar larvae. *BmTGIF* expression levels were analyzed as described in (B).

In *Drosophila*, *DmVis* is expressed abundantly in testis and ovary, and its expression profile indicates that it is present from embryogenesis to adulthood [32]. *BmTGIF*-*L* transcripts were expressed in various tissues, except midgut, and predominantly in testes, ovaries and hemocytes (Fig. 3A). Q-PCR showed that *BmTGIF*-*L* expression in the testis was higher in third instar larvae (Fig. 3B), when primary spermatocytes occupy most of the chamber of the testis, compared to other stages. In addition, the expression levels of the four isoforms of *BmTGIF* relative to *B. mori actin A3* in testes were estimated by Q-PCR, and *BmTGIF-MS1* demonstrated the highest expression level, being 3, 2 and 1.3 times higher than *BmTGIF-L*, *BmTGIF-S* and *BmTGIF-MS2*, respectively (Fig. 3C).

### BmTGIF-L Contains Two Acidic Activation Domains

**Figure 4:**
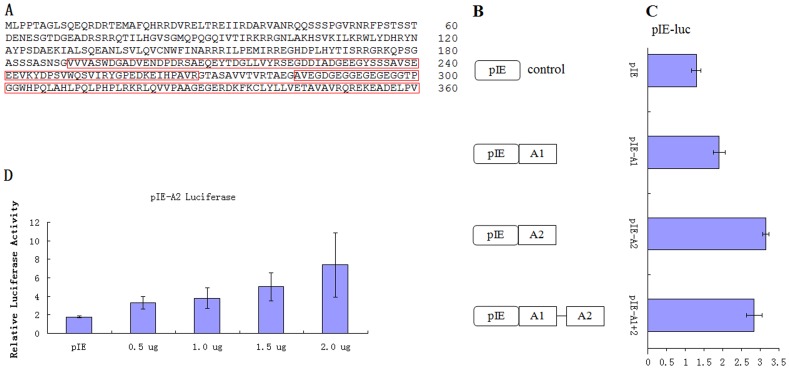
BmTGIF contains two acidic activation domains. (A) The two acidic regions are boxed within the full amino acid sequence of BmTGIF, the first acidic region is designated as A1, and the second acidic region is designated as A2. (B) A series of constructs containing acidic domains (homeodomain not included), that fused directly to downstream of BmNPV *ie-1* promoter of pIE were created. (C) The acidic domains constructs (2 µg) were cotransfected into BmN cells with the pIE-luc reporter (2 µg) which containing *luc* gene fused to 3′ end of BmNPV *ie-1* promoter in pIE plasmid, together with the control reporter plasmid pRL-TK (200 ng). The transfected cells were lysed for luciferase assay at 60 h post-transfection. Luciferase activity (mean ± standard deviation of triplicate transfections) is shown, together with the activity in cells expressing pIE alone. (D) pIE-luc reporter plasmid (1 µg) was cotransfected with pRL-TK (100 ng) and pIE-A2 (0.5, 1.0, 1.5, and 2.0 µg) constructs in BmN cells as indicated, the relative luciferase activity is shown in arbitrary units.

Human TGIF is a transcriptional repressor that effectively represses the transcriptional activity of several genes at their promoters [10], . In contrast to human TGIF, however, DmVis is a transcriptional activator [14]. It was therefore of interest to determine if BmTGIF was a transcriptional activator or repressor. A1 (at positions 190–268) and A2 (at positions 283–360), the two acidic regions (Fig. 4A) of BmTGIF-L, were assigned based on the similarity to the acidic regions in DmVis. A1 and A2 differed in the percentage of acidic residues; the percentage of acidic residues in A1 is higher than that in A2. We constructed luciferase assay plasmids pIE-A1, pIE-A2 and pIE-A1+2 (Fig. 4B) and tested the effect of the acidic regions on a luciferase reporter (pIE-luc). As shown in Fig. 4C, transfection of BmN cells with these constructs containing the acidic regions of *BmTGIF*-*L* together with the pIE-luc reporter increased transcriptional activation of the reporter compared with the pIE construct alone (Fig. 4C). Furthermore, luciferase activity elevated by pIE-A2 occurred in a dose-dependent manner (Fig. 4D). Together, these results demonstrated that BmTGIF-L contains two acid-rich transcriptional activation domains.

### Effect of *BmTGIF* siRNA Injection into Silkworm Larvae on the Development of Sperm Cells

**Figure 5:**
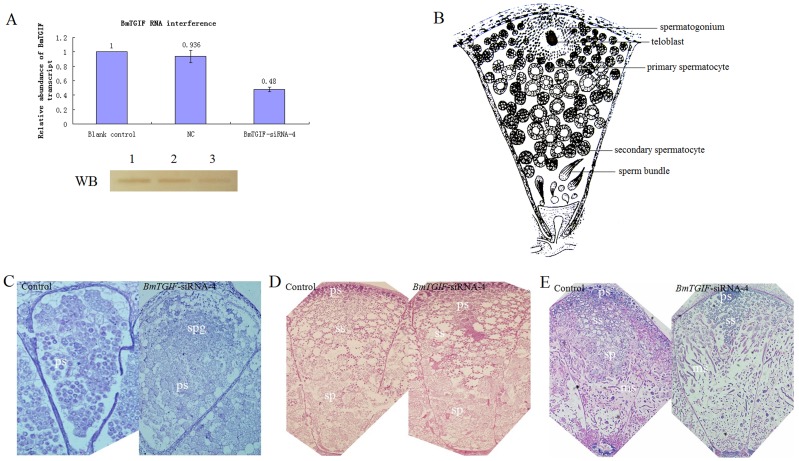
RNAi effect of *BmTGIF*-specific siRNA-4. (A) Effect of injecting *BmTGIF*-specific siRNA-4 into silkworm larvae at the beginning of the third, fourth and fifth instars. Relative amounts of *BmTGIF* mRNA in blank control, NC and *BmTGIF*-siRNA-4 groups were analyzed by Q-PCR at day 3 of fifth instar in triplicate, the *BmTGIF* mRNA level was normalized against the housekeeping cytoplasmic β−actin mRNA level. Lower panel represents BmTGIF protein detected in blank control, NC and *BmTGIF*-siRNA-4 group by western blotting using BmTGIF-L antibody, corresponding lanes marked by 1, 2, and 3, respectively. (B) A concise schematic showing the progression of spermatogenesis in *B. mori*. Spermatogonium born at the apical tip of first instar surround the teloblast, they undergo six rounds of synchronous mitotic divisions to produce cysts of 64 primary spermatocysts, the primary spermatocysts then enter an extended G2 phase characterized by a little increase in cell volume followed by the first meiotic division to produce larger cysts of secondary spermatocysts, which enter the second meiotic division resulting sperm bundle that containing mature sperms. (C), (D), and (E) represent the effects of RNAi on testis development in silkworms at the beginning of the fifth instar, the end of the fifth instar, and 10 days after pupation, respectively. spg, spermatogonium; ps, primary spermatocytes; ss, secondary spermatocytes; sp, cysts of spermatids; ms, mature sperms formed in sperm bundle. Original magnification = ×40.

In order to understand the functions of the *BmTGIF* gene, *BmTGIF*-specific siRNA (*BmTGIF*-siRNA-4) located at exon 3 of *BmTGIF*-L, was injected into silkworm larvae at the beginning of the third, fourth and fifth instars to silence the expression of the *BmTGIF* gene. The level of *BmTGIF*-L mRNA in the testes at the end of the fifth instar larvae was estimated by Q-PCR. The level of *BmTGIF-L* decreased by about 50% in the larvae injected with *BmTGIF*-siRNA-4 compared to that in the blank control and NC group (Fig. 5A). Moreover, The BmTGIF-L protein production in the larvae injected with *BmTGIF*-siRNA-4 was relatively lower than that in the blank control and NC group (Fig. 5A). We were therefore, interested to know if paraffin sections of testes injected with *BmTGIF*-specific siRNA showed the meiotic-arrest phenotype of testis development. Spermatogonium born at the apical tip of first instar surround the teloblast, they undergo six rounds of synchronous mitotic divisions to produce cysts of 64 primary spermatocysts, the primary spermatocysts then enter a extended G2 phase characterized by a little increase in cell volume followed by the first meiotic division to produce larger cysts of secondary spermatocysts, which enter the second meiotic division resulting sperm bundle that containing mature sperms (B). The development of sperm cells in the testes of larvae injected with *BmTGIF* siRNA-4 was later than that in blank controls (Fig. 5C and 5D). At the beginning of the fifth instar, the testis chamber in the blank control was full of larger primary spermatocytes organized into 64-cell cysts, indicating that meiotic division was progressing. In contrast, the testis in the *BmTGIF* siRNA-4 group was filled with large spermatogonia and smaller primary spermatocytes, a specialized stage that precedes meiotic divisions (Fig. 5C). Fig. 5D shows the results of histochemical staining of dissected testes at the end of the fifth instar. In the blank control group, primary spermatocytes had mostly developed into secondary spermatocytes, and many spermatids were observed far from the apical tip of the testes. In the testes of larvae injected with *BmTGIF* siRNA-4, however, a block of primary spermatocytes still remained near the cellula apicalis (Fig. 5D). This phenomenon was also observed at the pupal stage of larvae injected with *BmTGIF* siRNA-4 (Fig. 5E). As shown in Fig. 5E, a number of secondary spermatocytes (ss) developed more slowly than control group, the size of secondary spermatocyte cysts was obviously smaller compared to control group. Overall, these results suggest that progression of spermatid development could be arrested by repressing the expression of *BmTGIF*-L gene.

### Subcellular Localization of BmTGIF-L in BmN Cells and Testis

**Figure 6:**
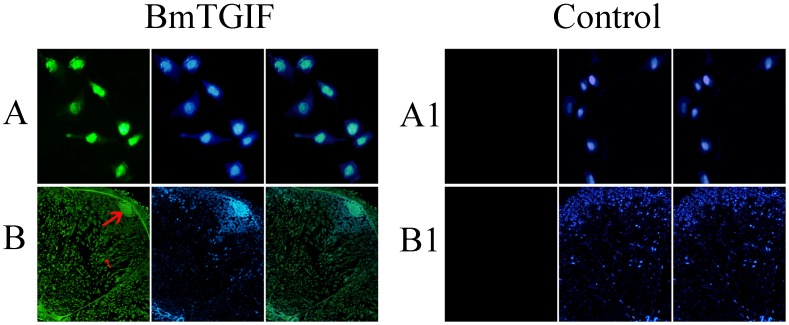
Immunofluorescent images of BmTGIF expression in BmN cells (A) and testis at 10 days after pupation (B). Immunostaining was performed with mouse anti-BmTGIF-L antibody, followed by treatment with FITC-conjugated goat anti-mouse IgG. The nuclei of BmN cells and testis were treated with DAPI (blue), and examined under an inverted fluorescence microscope. In both (A) and (B), from left to right, green fluorescence for FITC-treated BmTGIF protein, DAPI-treated nuclei, and the overlay images. The primary spermatocytes (large arrow) and mature sperm (small arrow) also showed prominent BmTGIF expression. Primary spermatocytes (large arrow) also showed strong DAPI labeling, overlaid with FITC staining. For controls, pre-immune serum was used as the primary antibody. Original magnification for BmN cells = ×200, for testis = ×40.

The results of immunofluorescence showed that BmTGIF-L in BmN cells was distributed mainly in the nucleus, overlaid with DNA (Fig. 6A), while it was expressed in primary spermatocytes and mature sperms in the testis of the pupal stage (Fig. 6B). DAPI labeling suggested that BmTGIF-L was tightly associated with DNA in primary spermatocytes (Fig. 6B). On the contrary, no green fluorescence was observed in BmN cells and testis using pre-immune serum.

### BmTGIF-L and BmAly Exist in a Complex in Testes

**Figure 7:**
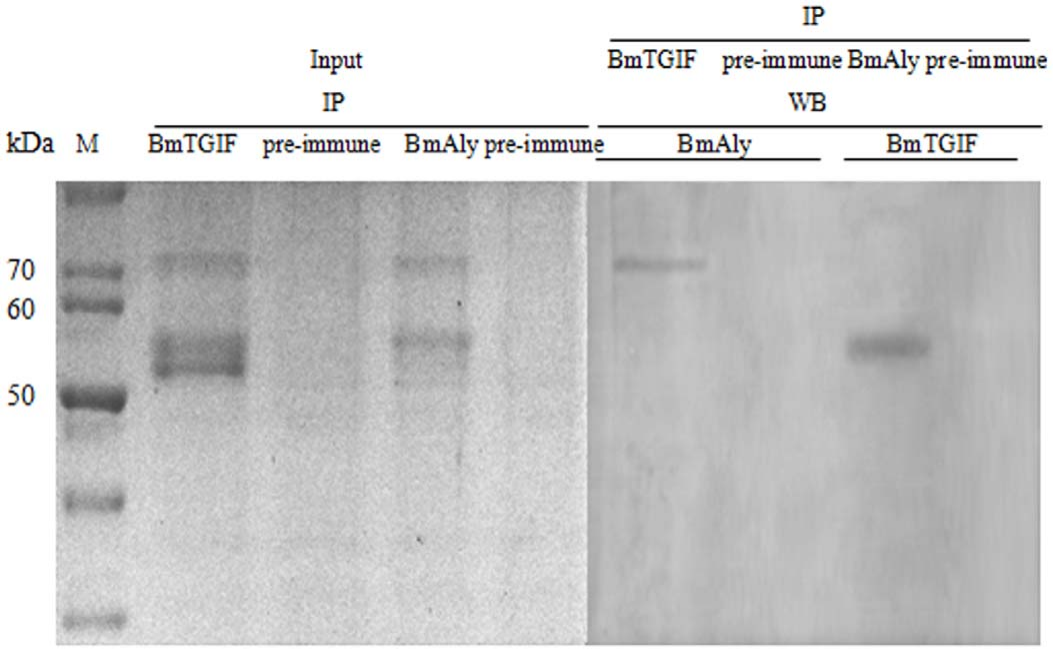
BmTGIF-L co-immunoprecipitated with BmAly. Extracts from wild-type testes were immunoprecipitated with anti-BmTGIF-L/pre-immune antiserum, run on SDS-PAGE gels, and subjected to Western blotting with anti-BmAly antibody, and vice versa. In the IP experiment using BmTGIF-L and BmAly anti body, BmAly and BmTGIF were observed at the specific bands, about 72 and 55 kDa, respectively. However, none of BmAly and BmTGIF-L were observed when IP using pre-immune antiserum. M, marker; input, input samples obtained from bombyx mori testes were immunoprecipitated with specific antibody in IP experiment, then run on SDS-PAGE; WB: western blotting for detecting the specific bands indicated.

Many proteins, including DmAly, DmComr, DmVis, DmTomb and DmTopi, are coexpressed in the nuclei of *Drosophila* primary spermatocytes where they work together to form a complex to regulate the activation of genes required for spermatid development [33], . To test if BmTGIF-L could interact with BmAly (GenBank accession no.GQ999610), a protein encoded by a meiotic-arrest gene in *B. mori*, immunoprecipitation using the anti-BmTGIF-L antibody was carried out to detect co-immunoprecipitation of BmAly and BmTGIF. As shown in Fig. 7, BmTGIF-L appeared to coprecipitate with BmAly. In the reciprocal experiment, immunoprecipitation with anti-BmAly antibodies and blotting with anti-BmTGIF-L also showed co-immunoprecipitation of BmAly and BmTGIF-L. In contrast, in control group using pre-immune antiserum, none of BmAly and BmTGIF-L was detected. These results suggest that these two proteins are present in a complex during spermatid development in *B. mori*.

## Discussion

TGIF has been shown to be a transcription factor, responsible for activating or repressing the transcriptional activation of target genes via interacting with co-activators or co-repressors [10], [14], [25], [27], [28], . The results of this study indicate that the *B. mori* homolog of DmTGIF, BmTGIF-L, has high similarity with TGIFs in the two conserved domains, and acts as a transcriptional coactivator of target genes. Furthermore, BmTGIF was located mainly in the nuclei of cultured BmN cells derived from ovary, and was highly expressed in primary spermatocytes. In addition, BmTGIF and BmAly may form a complex involved in regulating spermatogenesis.

In *Drosophila* spermatogenesis, *DmVis* has been classified into the group of meiotic-arrest genes, and interacts with other meiotic-arrest genes controlling meiotic divisions and spermatid differentiation. Mutation of *DmVis* resulted in failed transcriptional activation of many genes required for sperm manufacture and of some genes required for entry into meiotic divisions, causing arrest of testis development at the primary spermatocyte stage [14], [32], [33], . However, spermatogenesis in *B*. *mori* has been less well studied, though previous studies have identified an activator of Hsp90 ATPase (BmAHA1) [62] and a meiotic-arrest gene termed *Bmaly* (GenBank accession no.GQ999610).

Alignment of TGIF across a number of species suggests the existence of two highly conserved domains; the homeodomain and a 20-amino-acid block directly carboxy terminal to the homeodomain [14], [32]. The highest homologies are found within the homeodomain and the region carboxy-terminal to it in BmTGIF-L and DmVis. In particular, the characteristic RYN residues and the DNA-binding-determining residue within the conserved WF_N sequence [11] are identical, suggesting that BmTGIF-L may have the same characteristics, bind to the same DNA sequence, and perform similar functions to DmVis. Previous studies showed that *DmVis* was expressed from embryogenesis through to adulthood, and there were two alternative spliced products of *DmVis*. *Drosophila* testes predominantly express the larger (DmVisL), whereas ovaries predominantly express the shorter (DmVisS) [32]. In addition, DmVisL and DmVisS are required for *Drosophila* spermatogenesis, and transgenic flies containing either DmVisL or DmVisS are sufficient to partially rescue Df(2R)*pingpong*, which is a mutant lacking both *Dmachi* and *Dmvis* (a meiotic-arrest gene) [33]. The current study demonstrated that *BmTGIF-L* was also expressed in hemocytes and head, as well as in testis and ovary. Additionally, *B. mori* testes and ovaries expressed four and two alternatively-spliced products, respectively, from the *BmTGIF* transcription unit; whereas only the *BmTGIF*-L protein product was detected in testes and ovaries. The reason to interpret this maybe that: the lower titer of antibody of anti-BmTGIF to other isoforms, or the other spliced isforms can not be translated to proteins, or the detection limit of Western blotting, while the other alternative spliced isoforms were translated into few protein which is too less to be detected. In summary, these differed from the splicing pattern of *dTGIF*, suggesting that BmTGIF and dTGIF may have similar functions, but that BmTGIF may function at a large extent, and exert its action via more complicated mechanisms. Analysis of the phylogenetic tree could explain the functional difference between BmTGIF and dTGIF, which do not occur together in a subgroup. Recent studies of human TGIF have shown that only one isoform was abundantly expressed in various tissues and developmental stages, even though it possesses 12 alternatively-spliced isoforms, distinguished according to their 5′ cDNA sequences [63]. Additionally, reports of mouse TGIF2 indicate that an intron retained in the coding sequence of the second exon can be spliced out to produce two alternatively-spliced isoforms created by both alternate splicing and phosphorylation [60]. Interestingly, we found four alternatively-spliced variants (BmTGIF-L, BmTGIF-MS1, BmTGIF-MS2 and BmTGIF-S) in testes, and two (BmTGIF-L and BmTGIF-S) in ovaries. However, only BmTGIF-L had the conserved domain corresponding to dTGIF, suggesting that the other three isoforms did not have similar functions to dTGIF. Moreover, the expression levels of the alternative splicing forms were quite different. It is possible that this difference in expression levels between the spliced variants may contribute to their various functions, and the action of BmTGIF-L might be regulated by BmTGIF-MS1, BmTGIF-MS2 and BmTGIF-S, though further studies are needed to confirm this speculation.

The results of Q-PCR showed that *BmTGIF-L* was abundantly expressed in the testes of third instar larvae, when the testis is almost full of primary spermatocytes prior to the onset of meiotic divisions. The expression pattern of *BmTGIF-L* thus correlates well with that of *DmVis* [32], suggesting that *BmTGIF-L* may act at an earlier step like *DmVis* in the hierarchy of *B.mori* spermatogenesis. Moreover, BmTGIF was predominantly located in the nuclei of BmN cells, consistent with the functional state of DmVis. In contrast, immunostaining of BmTGIF in testes on day 10 of the pupal period strongly detected BmTGIF mRNA signals in mature sperms as well as primary spermatocytes, suggesting BmTGIF is present from pre- to postmeiotic stages, and could thus contribute to the regulation of meiotic divisions and post-meiotic differentiation, especially morphological changes of spermatids. Large amounts of DmVis, however, are transcribed before meiosis, and levels decrease dramatically at the post-meiotic stages [33].

Human TGIF and TGIF2 usually interact with corepressor proteins to repress the transcription of target genes [10], [27], [28], [58]. In contrast, *Drosophila* TGIFs have been reported to act as transcriptional activators that can activate gene expression whether bound to DNA directly, or tethered to DNA by the heterologous GBD [14]. Acidic activation domains are common in transcriptional regulators [64]. In the current study, luciferase reporter assays using constructs containing BmTGIF-L acidic domains suggested that the two acidic domains of BmTGIF-L might play the role of coactivator to enhance transcription.

Many meiotic-arrest genes have been reported in *Drosophila*, and these have been classified into two classes based on the mechanism by which they control the accumulation of twine. The *aly* class (including *aly*, *comr, achi/vis, topi* and *tomb*) regulates the transcription of twine, while the *can* class (including *can*, *mia* and *sa*) post-transcriptionally regulates twine production [33], . However, the *aly* class controls both the processes of meiotic divisions and spermatid differentiation. The mechanism by which they regulate meiotic cell cycle progression and cellular differentiation may be as follows: Achi/Vis, Topi and Tomb enter the nucleus independently, and Aly and Comr are imported into the nucleus together and form a stable complex. The complex then interacts with Tomb. Achi/Vis and Topi bind to the target promoters via specific DNA sequences, and later recruit Aly, Comr and Tomb to form an entire complex. This entire complex then recruits the NURD histone deacetylase chromatin remodeling complex and fully promotes transcription [43], [44]. During *B. mori* spermatogenesis, many factors must work together to trigger the male meiosis and spermatid differentiation programs. This speculation is partially supported by the co-immunoprecipitation of BmTGIF-L and BmAly by an anti- BmTGIF-L antibody, and vice versa. However, it is likely that unknown factors interact with BmTGIF-L and BmAly to form a complex to activate fully the programs of meiosis and spermatid differentiation in *B*. *mori*. Further studies are needed to clarify the mechanism responsible for controlling the meiosis and spermatid differentiation programs.

Mutations in *hTGIF* and *DmVis* cause HPE and arrest of *Drosophila* testis development at the primary spermatocyte stage, respectively. Our detailed examination of *BmTGIF*-RNAi silkworms showed an apparent decline of *BmTGIF* mRNA levels compared to blank controls and NC group though the protein production was relatively lower. In addition, paraffin sections of testes of larvae injected with *BmTGIF*-siRNA-4 exhibited developmental retardation, though this differed from the prominent arrest phenotype of *Drosophila* testis in the *pingpong* mutant. Taken together, these results suggest that BmTGIF-L regulates the progression of meiotic divisions and spermatid differentiation in *B.mori*, similar to the role played by DmVis in *Drosophila* spermatogenesis.
